# GLI family zinc finger protein 2 promotes skin fibroblast proliferation and DNA damage repair by targeting the miR‐200/ataxia telangiectasia mutated axis in diabetic wound healing

**DOI:** 10.1002/kjm2.12813

**Published:** 2024-02-22

**Authors:** Zun‐Hong Liang, Shi‐Shuai Lin, Zhi‐Yang Qiu, Yun‐Chuan Pan, Nan‐Fang Pan, Yun Liu

**Affiliations:** ^1^ Department of Burn & Skin Repair Surgery Hainan General Hospital (Hainan Affiliated Hospital of Hainan Medical University) Haikou P.R. China; ^2^ Department of Plastic and Cosmetic Surgery Hainan General Hospital, Hainan Affiliated Hospital of Hainan Medical University Haikou P.R. China

**Keywords:** ATM, DFU, DNA damage repair, GLI2, miR‐200 family

## Abstract

Diabetic foot ulcer (DFU) is a serious complication of diabetic patients which negatively affects their foot health. This study aimed to estimate the role and mechanism of the miR‐200 family in DNA damage of diabetic wound healing. Human foreskin fibroblasts (HFF‐1 cells) were stimulated with high glucose (HG). Db/db mice were utilized to conduct the DFU in vivo model. Cell viability was evaluated using 3‐(4,5‐dimethyl‐2‐thiazolyl)‐2,5‐diphenyl‐2‐H‐tetrazolium bromide assays. Superoxide dismutase activity was determined using detection kits. Reactive oxygen species determination was conducted *via* dichlorodihydrofluorescein‐diacetate assays. Enzyme‐linked immunosorbent assay was used to evaluate 8‐oxo‐7,8‐dihydro‐2′deoxyguanosine levels. Genes and protein expression were analyzed by quantitative real‐time polymerase chain reaction, western blotting, or immunohistochemical analyses. Luciferase reporter gene and RNA immunoprecipitation assays determined the interaction with miR‐200a/b/c‐3p and GLI family zinc finger protein 2 (GLI2) or ataxia telangiectasia mutated (ATM) kinase. HG repressed cell proliferation and DNA damage repair, promoted miR‐200a/b/c‐3p expression, and suppressed ATM and GLI2. MiR‐200a/b/c‐3p inhibition ameliorated HG‐induced cell proliferation and DNA damage repair repression. MiR‐200a/b/c‐3p targeted ATM. Then, the silenced ATM reversed the miR‐200a/b/c‐3p inhibition‐mediated alleviative effects under HG. Next, GLI2 overexpression alleviated the HG‐induced cell proliferation and DNA damage repair inhibition via miR‐200a/b/c‐3p. MiR‐200a/b/c‐3p inhibition significantly promoted DNA damage repair and wound healing in DFU mice. GLI2 promoted cell proliferation and DNA damage repair by regulating the miR‐200/ATM axis to enhance diabetic wound healing in DFU.

**Abbreviations**
8‐oxo‐dG: 8‐oxo‐7,8‐dihydro‐2′‐deoxyguanosine
ATM: ataxia telangiectasia mutated
ATR: ATM and Rad3‐related
DCFH‐DA: dichlorodihydrofluorescein‐diacetate
DDR: DNA damage response
DFU: diabetic foot ulcer
DMEM: Dulbecco's modified Eagle's medium
DNA‐PKcs: DNA‐dependent protein kinase catalytic subunit
ELISA: enzyme‐linked immunosorbent assay
ER: endoplasmic reticulum
GLI2: GLI family zinc finger protein 2
HG: high glucose
IHC: immunohistochemical analysis
MA: mannitol
miR‐200: microRNA‐200
MTT: 3‐(4,5‐dimethyl‐2‐thiazolyl)‐2,5‐diphenyl‐2‐H‐tetrazolium bromide
NG: normal glucose
PBS: phosphate buffer saline
qRT‐PCR: quantitative real‐time polymerase chain reaction
RIP: RNA immunoprecipitation
ROS: reactive oxygen species
SOD: superoxide dismutase
UPR: unfolded protein response

## 1 INTRODUCTION

Diabetic foot ulcer (DFU), a severe complication of diabetes, accounts for 5.3%–10.5% of all complications.^1^ DFU has been recognized as the leading cause of hospitalization, amputation, and mortality in patients with diabetes.^2^ Currently, treatment options for DFU have been focused on wound dressing, debridement, antibiotic treatment, and revascularization,^3^ whereas their efficacy and stability still require amelioration. Diabetic wound healing is known to play a vital role in the recovery of DFU, and the hyperglycemia of DFU is accompanied by significant delays in wound healing.^4^ Skin fibroblasts could exert critical effects on wound healing, such as the concentration of the wound.^5^ In diabetic hyperglycemia, oxidative stress and DNA damage increase, while DNA damage repair and cell survival decrease. Also, the precise mechanism of DNA damage repair in DFU skin fibroblasts remains elusive.

DNA belongs to the set of inherently active molecules that are highly sensitive to chemical modification.^6^ Aberrant or excessive DNA damage processes can promote apoptosis and exert detrimental effects on cell survival. Furthermore, a dedicated DNA damage response (DDR) mechanism protects genomic integrity and repairs DNA damage.^7^ The DDR network consists of DNA damage signal sensors, transducers, and effectors. Also, transducers' constitutions include the ataxia telangiectasia mutated (ATM) kinase, ATM, and Rad3‐related (ATR) kinase, and DNA‐dependent protein kinase catalytic subunit kinase in mammalian cells.^8^ Distinctively, ATM is the core regulator in DDR, playing a prominent role in double‐stranded DNA break repair.^9^ Cumulative experimental evidence has indicated that DNA damage is tightly involved in metabolic disorders, including diabetes mellitus and its complications. As demonstrated, enhanced DNA damage and repressed DNA repair have been found in DFU.^10^ Nevertheless, the detailed cellular mechanism of DNA damage repair and ATM in the skin fibroblasts of DFU has not been fully elucidated.

MicroRNAs (miRNAs) belong to the set of endogenous RNAs, which are vital parts of epigenetic mechanisms. MiRNAs can induce mRNA degradation and protein translation inhibition to modulate the progression of diseases (including diabetes and its complications).^11^ The miR‐200 family (including miR‐200a, 200b, 200c, 141, and 429) was markedly increased in the islet of the diabetic mouse, and the overexpressed miR‐200 family promoted diabetic mouse death.^12^ The downregulated miR‐200 family may promote diabetic wound healing by the enhancement of feasible angiogenesis.^13^ However, its precise role in the wound‐healing process of DFU remains unclear. Furthermore, miR‐200c inhibition could promote DNA damage repair in human keratinocytes under radiation.^14^ However, the miR‐200 family's function in the DNA damage repair of DFU's wound healing remains unintelligible. In addition, based on our bioinformatics prediction, there may be potential binding sites between the miR‐200 family (miR‐200a/b/c) and ATM. Their interaction in the DNA damage of skin fibroblasts needs to be explored further for the potential target selection of DFU.

Sonic hedgehog (Shh) signaling is commonly regarded as a classic pathway in the tissue repair and regeneration process. The significant inhibition of Shh signaling has been identified in type 1 diabetes mellitus, and its activation can enhance the cellular function of injured endothelial progenitor cells to promote diabetes cardiac repair.^15^ Shh signaling activation includes interacting with their patched receptor (patched 1), relieving the inhibitive receptor (smoothened frizzled class receptor), and activating the glioma‐associated oncogene homolog (GLI) transcription factor members (such as GLI2).^16^ Decreased GLI2 has also been found in the diabetic animal and high glucose (HG)‐stimulated cell model.^17^ Moreover, the Shh protein can promote skin wound healing via the inhibition of the miR‐200 family in embryonic stem cells.^18^ Also, from the prediction performed using hTFtarget and the JASPAR database, we found that GLI2 may possess potential binding sites in the promoter region of the miR‐200 family. However, their detailed mechanisms in diabetic wound healing and DNA damage of skin fibroblasts have not been investigated currently.

In this study, we used diabetic mice and human foreskin fibroblasts (HFF‐1 cells) to investigate the cellular mechanism of DNA damage in skin fibroblasts for DFU wound healing. We supposed that overexpressed GLI2 might downregulate the miR‐200 family members, thereby regulating ATM expression and further affecting the DNA damage repair process of skin fibroblasts. Studying this mechanism will lead to the discovery of more effective therapies for the promotion of wound healing in DFU patients.

## 2 MATERIALS AND METHODS

### 2.1 Cell culture and treatment

HFF‐1 cells were acquired from the American Type Culture Collection (Manassas, VA, USA). HFF‐1 cells were grown in Dulbecco's modified Eagle's medium (Sigma‐Aldrich, MO, USA) containing 15% fetal bovine serum (Gibco, Carlsbad, CA, USA) and penicillin/streptomycin (100 U/mL/0.1 mg/mL). The cells were maintained at 37°C in a humidified incubator and with 5% CO_2_ and 95% air. For the establishment of a DFU in vitro model, HFF‐1 cells were stimulated with HG of various concentrations (0, 5, 10, 20, 30, 50, and 100 mM) for 24 h based on a previous study.^19^ The final glucose treatment concentration was selected for formal detection according to the cell viability and injury reactions. There were three groups of HFF‐1 cells; the normal glucose group (NG, 5 mM), the HG group, and the mannitol group (MA, using the same concentration of HG to avoid the effect of highly osmotic factors). After the treatment, HFF‐1 cells were administered the relevant overexpression or inhibition transfection (ATM, GLI2, and miR‐200).

### 2.2 Cell transfection

To overexpress or inhibit miR‐200a/b/c‐3p, the matching miR‐200a/b/c‐3p mimics, inhibitors, and the corresponding negative control (mimics NC or inhibitor NC) were obtained from Genepharma (Shanghai, China). Also, the small interfering RNAs (siRNAs) targeting ATM and relevant si‐NC were offered by Genepharma. Moreover, relevant full‐length cDNAs were obtained from Genepharma and subcloned into the pcDNA3.1 vectors (Invitrogen, Carlsbad, CA, USA) to conduct pcDNA3.1‐GLI2 vectors. HFF‐1 cells were transfected with the above by the Lipofectamine 2000 (Invitrogen, Carlsbad, CA, USA) per the kits' detailed guidelines.

### 2.3 DFU mice model establishment

The spontaneous type 2 diabetes db/db mice (*n* = 36, male, 6–7 weeks old, 25–30 g) and non‐diabetic db/m mice (*n* = 36, male, 6–7 weeks old, 25–30 g) were acquired from the Beijing HFK Bioscience (Beijing, China). The mice were supplied with rodent standard feed and water ad libitum. All the mice were kept at 22°C ± 1, 50% ± 10 humidity conditions, and a 12‐h light/dark cycle. The blood glucose level was measured using a glucometer (Roche, Lyon, France). When the blood glucose was above 16.7 mmol for three consecutive days, the diabetic mice were successfully induced. Excisional wounds (5 mm × 5 mm) were created on the foot skin of the treatment or control mice which received inhaled isoflurane (2%) anesthetization after the diabetic mouse model establishment. Foot wound tissues were treated with antagomiR‐200a/b/c‐3p, NC, and smeared the glucagon‐like peptide 1 analog, liraglutide (100 nM) to investigate their mechanisms of diabetic wound healing.^20^ All experimental procedures were conducted per the ethical standards of the Experimentation Ethics Committee.

### 2.4 3‐(4,5‐Dimethyl‐2‐thiazolyl)‐2,‐diphenyl‐2‐H‐tetrazolium bromide assays

HFF‐1 cells (1 × 10^4^ cells/well) were seeded and cultured in 96‐well plates for 48 h. Thereafter, 3‐(4,5‐dimethyl‐2‐thiazolyl)‐2,5‐diphenyl‐2‐H‐tetrazolium bromide solution (20 μL, 5 mg/mL, Solarbio Biotech, Beijing, China) was supplemented in every well and further incubated for 4 h, after which dimethyl sulfoxide (100 μL) was added to the well. The absorbance was read by the SuPerMax 3000AL microplate reader (Shanpu Biotech, Shanghai, China) at 490 nm.

### 2.5 Reactive oxygen species determination

The reactive oxygen species (ROS) levels were determined using dichlorodihydrofluorescein‐diacetate (DCFH‐DA) reagents (Beyotime Biotech, Beijing, China). Briefly, HFF‐1 cells were incubated with the DCFH‐DA fluorescent probe (10 μM) for 20 min at 37°C in the serum‐free medium. Cells were then washed with a serum‐free medium to remove extracellular DCFH‐DA. The abovementioned cells were observed using a fluorescence microscope (Leica Microsystems, Wetzlar, Germany), based on the previous ROS measurement protocol.

### 2.6 Superoxide dismutase detection assays

The Total Superoxide Dismutase Assay Kit with NBT (Beyotime Biotech, Beijing, China) was used to measure the intracellular superoxide dismutase (SOD) levels. HFF‐1 cells (4 × 10^4^ cells/well) were collected, ice‐homogenized, and centrifugated to obtain the cell supernatant. The 20 μL of cell supernatant and 180 μL of prepared detection SOD solution were supplemented to the 96‐well plate and incubated for 30 min at 37°C, per the kit's protocol. The absorbance was read using the microplate reader (Shanpu Biotech, Shanghai, China) at 560 nm.

### 2.7 Enzyme‐linked immunosorbent assay

The DNA oxidative damage marker 8‐oxo‐7,8‐dihydro‐2′‐deoxyguanosine (8‐oxo‐dG) in the culture supernatant was evaluated using enzyme‐linked immunosorbent assay kits, per the kits' instructions (Elabscience Biotechnology, Wuhan, China). The corresponding absorbance was read on the SuPerMax 3000AL microplate reader (Shanpu Biotech, Shanghai, China) at 450 nm.

### 2.8 Quantitative real‐time polymerase chain reaction

Total RNA was isolated from cells and mice foot wound skin tissues using the MolPure Cell/Tissue miRNA Kit (Yeasen Biotech, Shanghai, China). Thereafter, the reverse transcription of miRNA to cDNA and further miRNA determination was conducted using the miRcute Plus miRNA First‐Strand cDNA kit (Tiangen Biotech, Beijing, China) and miRcute miRNA qPCR kit (SYBR green, Tiangen Biotech), per kits' appendant protocols. Also, the relative miRNA levels were evaluated and analyzed by the 7900HT Fast Real‐Time PCR System (Applied Biosystems, Waltham, MA, USA). U6 mRNA was chosen as a control to normalize the miR‐200a/b/c‐3p expression. The 2^−∆∆Ct^ method was employed to calculate the relative changes of miR‐200a/b/c‐3p to the control. The primer sequences are listed below:

miR‐200a‐3p‐F: 5′‐GGCACTGTCTGGTAACGATGTAA‐3′, miR‐200a‐3p‐R: 5′‐TGGTGTCGTGGAGTCG‐3′;
miR‐200b‐3p‐F: 5′‐TGCGGGTGCTCCGCTCCGCAGC‐3′, miR‐200b‐3p‐R: 5′‐GTGCAGGGTCCGAGGT‐3′;
miR‐200c‐3p‐F: 5′‐ACACTCCAGCTGGGTAATACTGCCGGGTAAT‐3′, miR‐200c‐3p‐R: 5′‐TGGTGTCGTGGAGTCG‐3′;
h‐ATM‐F: 5′‐GCCATATGTGAGCAAGCAGC‐3′, h‐ATM‐R: 5′‐CGAAGAACAAAGGCCCAAGC‐3′;
GAPDH‐F: 5′‐CCAGGTGGTCTCCTCTGA‐3′, GAPDH‐R: 5′‐GCTGTAGCCAAATCGTTGT‐3′;
U6‐F:5′‐CTCGCTTCGGCAGCACA‐3′, U6‐R: 5′‐AACGCTTCACGAATTTGCGT‐3′.

### 2.9 Western blot analysis

The total protein of cells and mice foot wound skin tissues were extracted using the Total Protein Extraction Kits (Thermo Fisher, Waltham, MA, USA). The total protein concentration was examined with a Bicinchoninic Acid Protein Assay Kit (BCA, Solarbio Biotech, Beijing, China). After that, the equal protein samples (20 μg) were separated using 12% sodium dodecyl sulfate‐polyacrylamide gel electrophoresis and electrotransferred to polyvinylidene difluoride membranes (PVDF, Millipore, Billerica, MA, USA) for 12 h at 4°C. Then, the PVDF membranes were blockaded with a 5% Bovine Serum Albumin Blocking Buffer (BSA, Solarbio Biotech, Beijing, China) for 1 h. Thereafter, the membranes were incubated with the primary antibodies for 12 h at 4°C. Afterward, the relevant secondary horseradish peroxidase (HRP)‐conjugated antibodies were used for the 1‐h incubation of the membranes at room temperature. Protein bands' chemiluminescence signals were captured using the ECL Western Blotting Substrate (Solarbio biotech) on the ImageQuant LAS system (GE Healthcare, Sunnyvale, CA, USA). The primary antibodies, including ATM (1:1000, no. 27156‐1‐AP, Proteintech, Rosemont, IL, USA) and GLI2 (1:1000, no. 18989‐1‐AP, Proteintech), were purchased from Proteintech. GAPDH (1:5000, no. 5174, CST, Beverly, MA, USA) was chosen as the normalization control.

### 2.10 RNA immunoprecipitation assay

Interactions between miR‐200a/b/c‐3p and ATM were verified using the Magna RNA immunoprecipitation (RIP) kit (Millipore, Billerica, MA, USA). HFF‐1 cells were lysed in the RIP lysis buffer, and then, the cell extraction (20 μg protein) was incubated containing magnetic beads bounded with antibodies anti‐Argonaute2 (Ago2, Millipore) and normal IgG (Millipore) for 4 h, at 4°C. The precipitate complex was digested using the Proteinase K buffer, and the coimmunoprecipitated RNA of the complex was isolated with the MolPure Cell/Tissue miRNA Kit (Yeasen Biotech). Thereafter, the relative expression levels of miR‐200a/b/c‐3p were evaluated by quantitative real‐time polymerase chain reaction (qRT‐PCR) analysis.

### 2.11 Luciferase reporter assay

The predicted binding sites of miR‐200a/b/c‐3p on ATM (WT‐ATM) or GLI2 (WT‐GLI2) and their mutated site (MUT‐ATM or MUT‐GLI2) were cloned into the firefly luciferase gene in PGL3 vector (Promega, Madison, WI, USA). HFF‐1 cells were cultured in 24‐well plates for 12 h, then the cells were cotransfected with the WT‐ATM/MUT‐ATM or WT‐GLI2/MUT‐GLI2 reporter gene plasmid and miR‐200a/b/c‐3p mimics or mimics NC. The Dual‐Luciferase Reporter Assay System (Promega) was utilized to evaluate luciferase activities after a 48‐h incubation period.

### 2.12 Immunohistochemical analysis

Thin sections (4 μm) of mice foot wound skin tissues were acquired from formalin‐treated and paraffin‐embedded tissues. Sections were conducted with dewaxing in xylene, rehydration, and washing in PBS. Thereafter, sections were administered antigen retrieval using Tris‐EDTA buffer (pH: 9.0), and the removal of endogenous peroxidase activity was performed by incubating in hydrogen peroxide solution (3%). After blocking for 45 min with 5% BSA and washing with PBS, sections were incubated with primary antibodies, including ATM (1:200, no. 27156‐1‐AP, Proteintech, Rosemont, IL, USA), for 12 h at 4°C. Then, sections were incubated with HRP‐labeled secondary antibodies for 1 h at 25°C. Further, washed sections were incubated with diaminobenzidine tetrahydrochloride for 5 min at 25°C. Finally, the microscope (Leica Microsystems, Wetzlar, Germany) and ImageJ software (NIH, Bethesda, MA, USA) were used to observe and count the stained regions of sections. The above procedure was based on the experimental protocol of a previous study.^21^

### 2.13 Statistical analysis

Statistical analyses were performed using SPSS 20.0 software (SPSS, Inc., Chicago, IL, USA). Multiple‐group comparisons were performed using the one‐way analysis of variance (ANOVA), followed by Tukey's test. Quantitative data were presented as the mean ± standard deviation, while categorical data were presented as frequencies and percentages. All experiments of this study were performed in triplicate independently. The threshold for statistical significance was set at *p* < 0.05.

## 3 RESULTS

### 3.1 HG repressed cell proliferation and DNA damage repair in HFF‐1 cells

**FIGURE 1:**
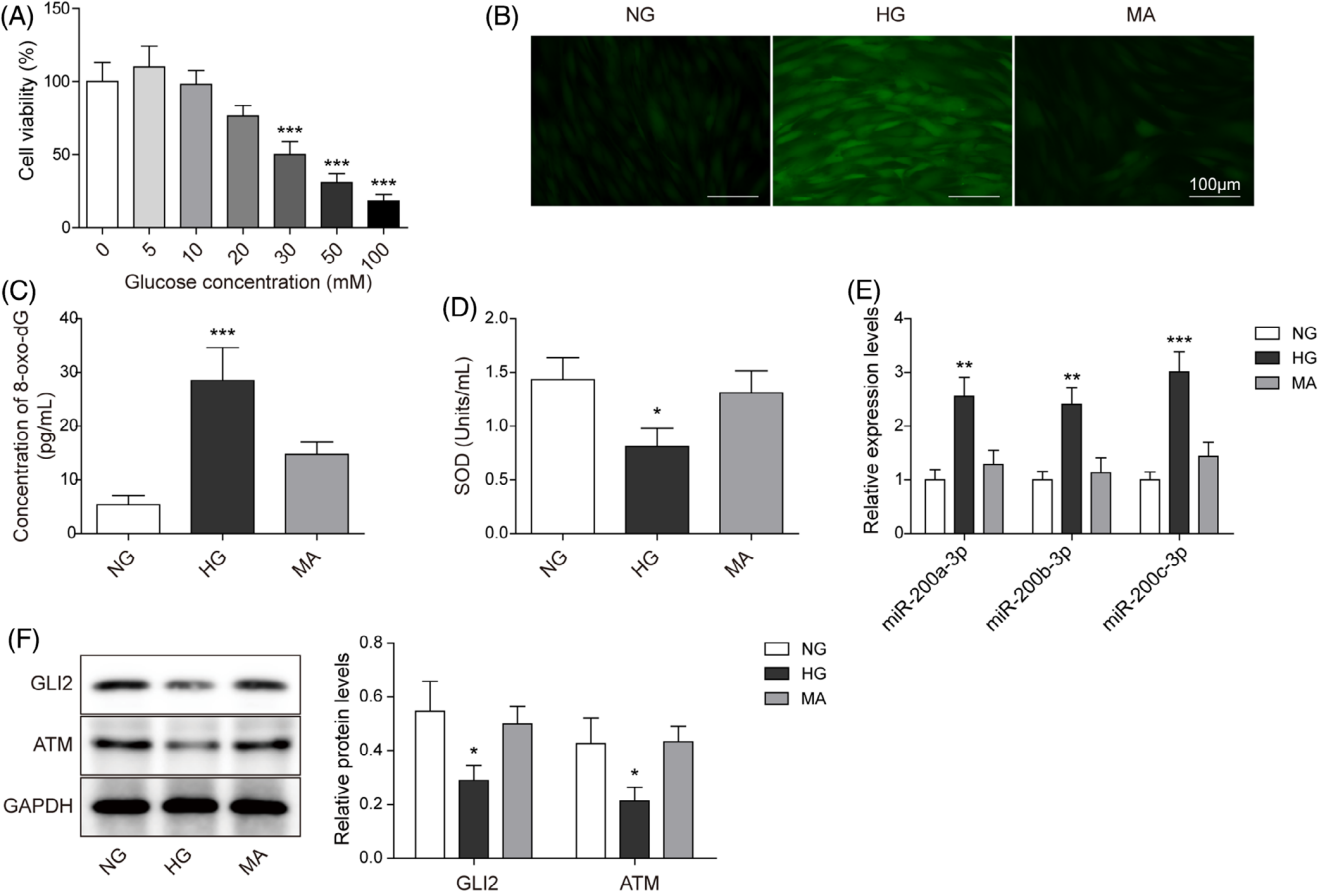
High glucose (HG) repressed cell proliferation and DNA damage repair in human foreskin fibroblasts (HFF‐1 cells). HFF‐1 cells were stimulated with normal glucose (NG), mannitol (MA), and HG. (A) 3‐(4,5‐dimethyl‐2‐thiazolyl)‐2,5‐diphenyl‐2‐H‐tetrazolium bromide assays estimated cell viability. (B) Dichlorodihydrofluorescein‐diacetate assays determined ROS levels (Scale bar = 100 μm). (C) Enzyme‐linked immunosorbent assay assays evaluated 8‐oxo‐7,8‐dihydro‐2′deoxyguanosine (8‐oxo‐dG) levels. (D) Superoxide dismutase (SOD) activity was measured using SOD detection assays. (E) Quantitative real‐time polymerase chain reaction detected the relative expression of miR‐200a/b/c‐3p. (F). Western blot detected the expression of GLI family zinc finger protein 2 (GLI2) and ataxia telangiectasia mutated (ATM). Each experiment was independently repeated three times. *p < 0.05, **p < 0.01, ***p < 0.001.

To explore the detrimental effects of diabetic hyperglycemia on skin fibroblasts, we established an HG‐stimulated in vitro injury model of HFF‐1 cells. The cells were stimulated with glucose (0, 5, 10, 20, 30, 50, and 100 mM) for 24 h, based on the previous protocol.^19^ As described in (Figure 1A), HG significantly reduced cell viability in a dose‐dependent manner. Based on the calculation of the IC_50_ value of glucose, we selected the 30 mM glucose concentration as the subsequent HG treatment concentration and used MA treatment (30 mM) to avoid the hypertonic effects of glucose on the cell injury reaction. HG treatment markedly increased the ROS levels, compared with the NG and MA group (Figure 1B). In keeping with the enhanced oxidative stress trend, 8‐oxo‐dG levels were significantly upregulated by HG stimulation (Figure 1C). HG also decreased SOD activities (Figure 1D). Additionally, miR‐200a/b/c‐3p expression was markedly enhanced by HG treatment (Figure 1E). Furthermore, GLI2 and ATM protein expression in HG groups were significantly lower than that of the NG and MA groups (Figure 1F). Overall, these data demonstrated that HG treatment repressed the cell proliferation and DNA damage repair, whereas it promoted the miR‐200a/b/c‐3p expression in HFF‐1 cells.

### 3.2 Inhibiting miR‐200 alleviated the HG‐induced repression of DNA damage repair

**FIGURE 2:**
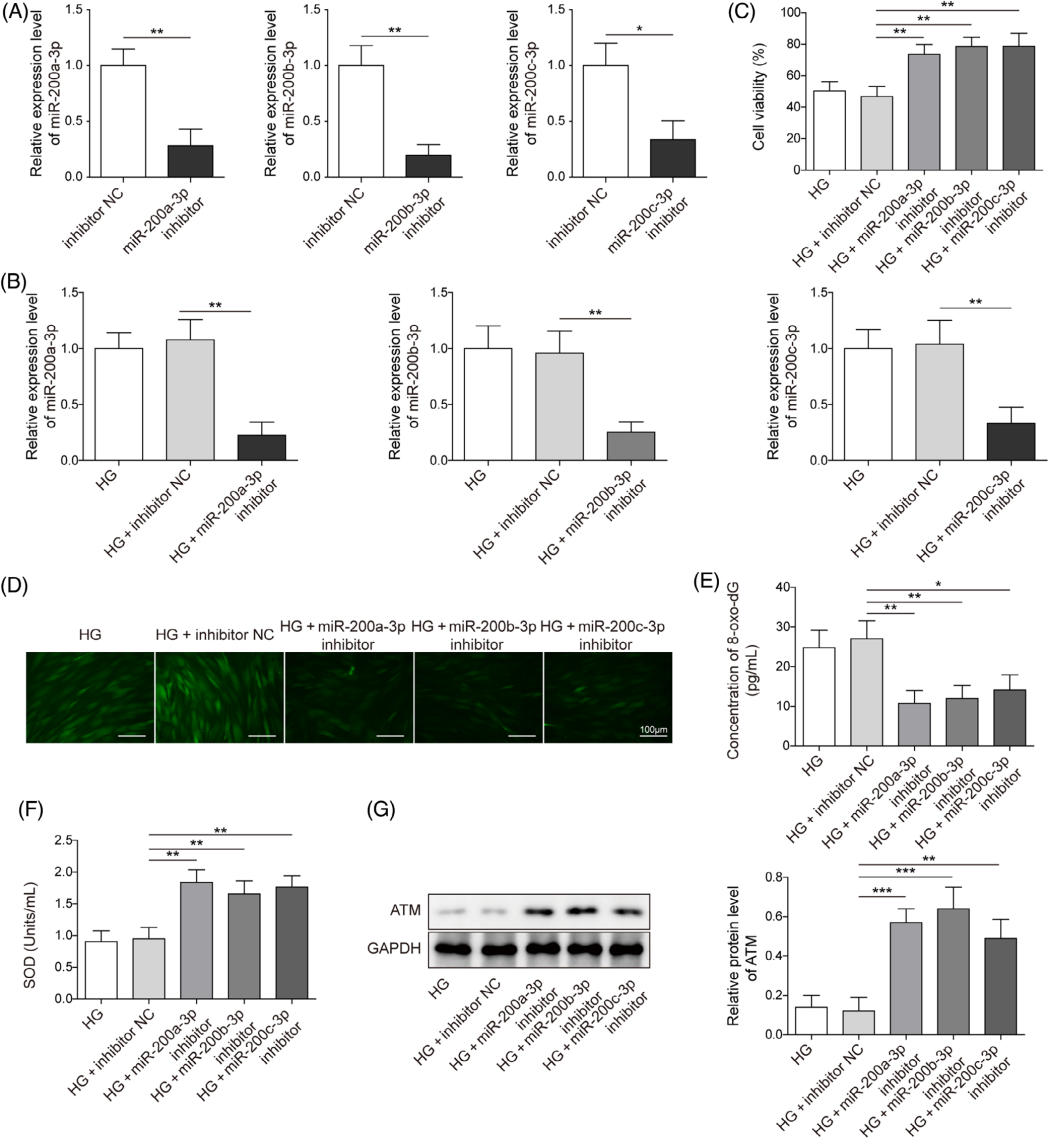
The MiR‐200 inhibitor alleviated the high glucose (HG)‐induced repression of DNA damage repair. Human foreskin fibroblasts were treated with HG and transfected with the miR‐200a/b/c‐3p inhibitor and inhibitor negative control (NC). (A) Quantitative real‐time polymerase chain reaction (qRT‐PCR) detected the expression of miR‐200a/b/c‐3p under normal glucose (NG) conditions. (B) qRT‐PCR detected the relative expression of miR‐200a/b/c‐3p under HG conditions. (C) 3‐(4,5‐dimethyl‐2‐thiazolyl)‐2,5‐diphenyl‐2‐H‐tetrazolium bromide assays estimated cell viability. (D) Dichlorodihydrofluorescein‐diacetate assays determined the ROS levels (Scale bar = 100 μm). (E) Enzyme‐linked immunosorbent assay (ELISA) assays evaluated 8‐oxo‐7,8‐dihydro‐2′deoxyguanosine (8‐oxo‐dG) levels. (F) Superoxide dismutase (SOD) activity was measured by SOD detection assays. (G). Western blot detected ataxia telangiectasia mutated (ATM) expression. Each experiment was independently repeated three times. *p < 0.05, **p < 0.01, ***p < 0.001.

To examine the function of the miR‐200 family in the HG‐induced DNA repair repression, the inhibition vectors targeting them were transfected to HFF‐1 cells. As displayed in (Figure 2A), miR‐200a/b/c‐3p expression was significantly decreased in NG‐treated HFF‐1 cells of the inhibitor transfection groups compared with the NC groups, suggesting the successful establishment of the miR‐200a/b/c‐3p inhibition cell model. Also, the inhibitor transfection markedly downregulated miR‐200a/b/c‐3p expression under HG conditions (Figure 2B). Moreover, the miR‐200a/b/c‐3p inhibitors reversed the cell viabilities decline of HFF‐1 cells stimulated with HG (Figure 2C). HG‐induced oxidative stress characterized by high levels of ROS was alleviated by miR‐200a/b/c‐3p inhibition (Figure 2D). Therefore, the levels of the HG‐increased DNA damage marker, 8‐oxo‐dG, were repressed by miR‐200a/b/c‐3p inhibitors (Figure 2E). However, miR‐200a/b/c‐3p inhibition alleviated HG‐induced decrease of SOD activity (Figure 2F). Additionally, miR‐200a/b/c‐3p inhibition significantly promoted the expression of the DNA damage repair protein, ATM (Figure 2G). Experimental evidence revealed that the miR‐200a/b/c inhibition could ameliorate cell proliferation and DNA repair suppression under HG conditions.

### 3.3 MiR‐200 targeted ATM and downregulated its expression

**FIGURE 3:**
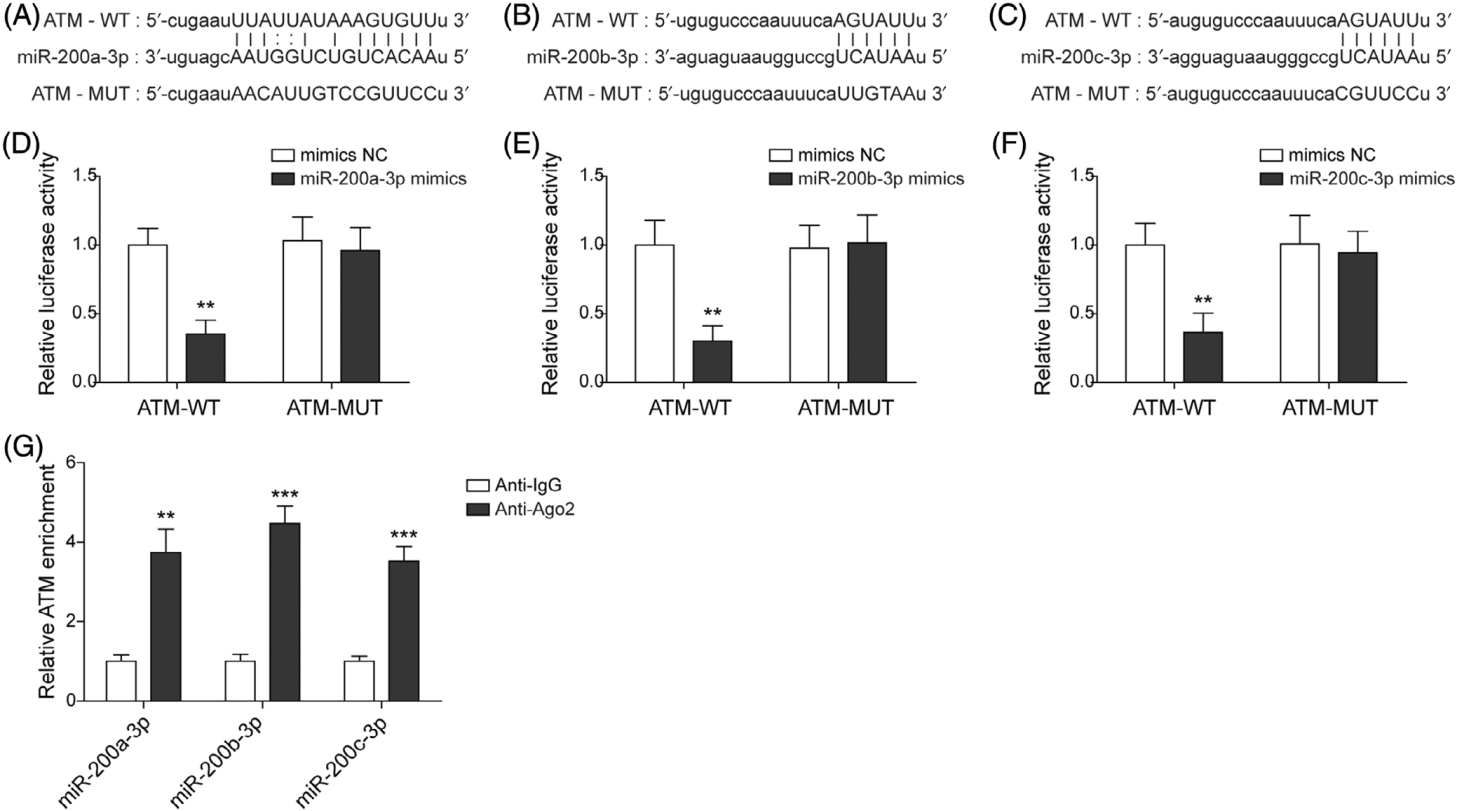
MiR‐200 targeted ataxia telangiectasia mutated (ATM) and downregulated its expression. Human foreskin fibroblasts were transfected with miR‐200a/b/c‐3p mimics and mimics NC. (A–C) Bioinformatics predicted the binding sites between ATM and miR‐200a/b/c‐3p. (D–F) Dual‐luciferase reporter assays were used to determine the interaction between ATM and miR‐200a/b/c‐3p. (G) The interaction between ATM and miR‐200a/b/c‐3p was validated by RNA immunoprecipitation assays. Each experiment was independently repeated three times. *p < 0.05, **p < 0.01, ***p < 0.001.

To investigate the modulatory mechanism between the miR‐200 family and ATM, we explored the existence of any interactions between them. From the online bioinformation prediction (http://starbase.sysu.edu.cn/), we found miR‐200a/b/c‐3p possessed potential binding sites on ATM (Figure 3A–C). The corresponding luciferase reporter assay results indicated that the activities of luciferase were downregulated by miR‐200a/b/c‐3p mimics in the ATM‐WT‐containing cells, whereas there was no obvious change in the ATM‐MUT‐transfected groups (Figure 3D–F). Furthermore, the RIP analysis also validated that miR‐200a/b/c‐3p possessed binding sites in the 3′UTR region of ATM, and the relative enrichment of ATM was significantly higher in the anti‐Ago2 groups than in the anti‐IgG groups (Figure 3G). In summary, this section of the results revealed that miR‐200a/b/c‐3p could target ATM and repress its expression.

### 3.4 ATM silencing reversed the regulation of miR‐200 inhibition in DNA damage repair under HG

**FIGURE 4:**
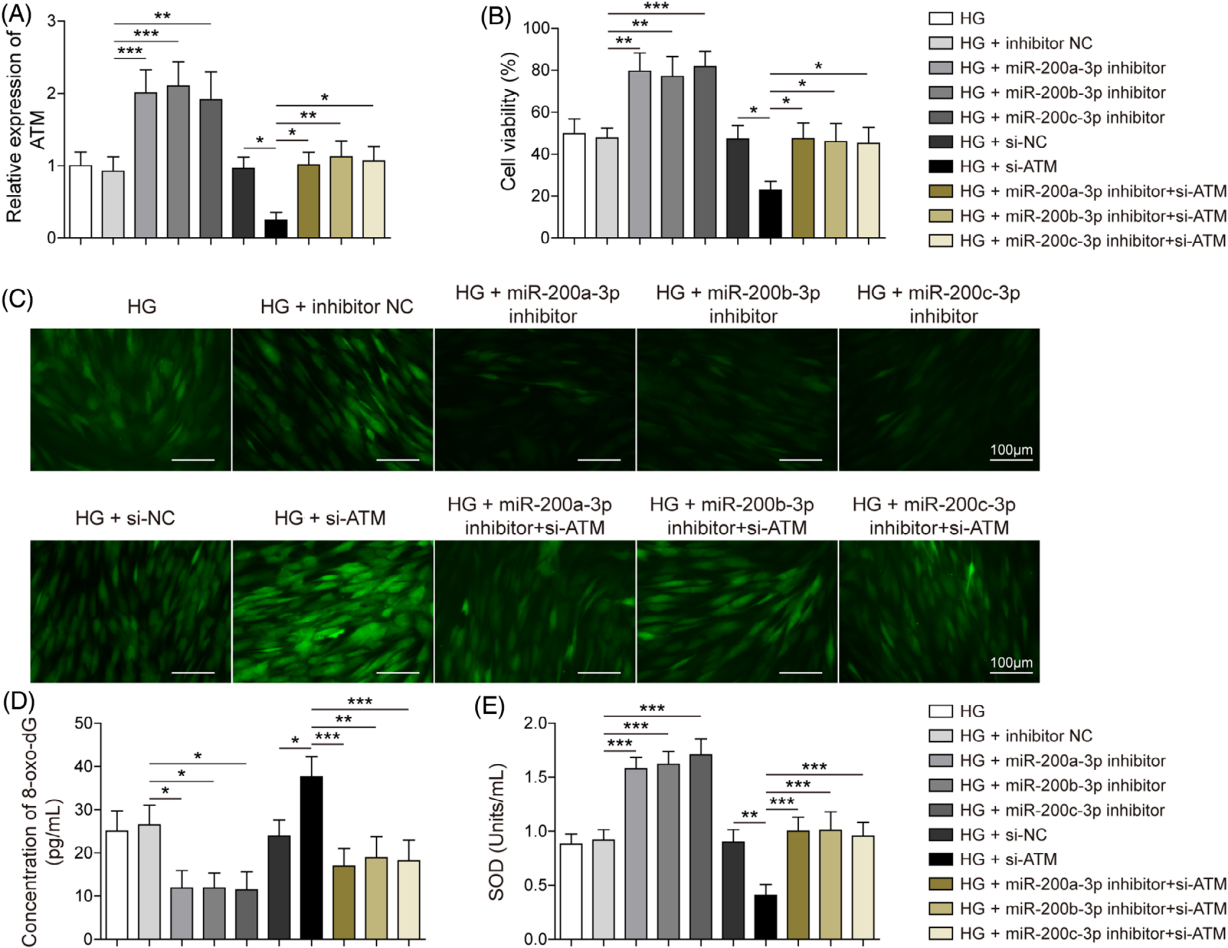
Ataxia telangiectasia mutated (ATM) silencing reversed the regulation of the miR‐200 inhibitor in DNA damage repair under HG conditions. Human foreskin fibroblasts were treated with high glucose (HG) and transfected with the miR‐200a/b/c‐3p inhibitor, inhibitor negative control (NC), si‐ATM, and si‐NC. (A) Quantitative real‐time polymerase chain reaction assays the detected ATM expression. (B) 3‐(4,5‐dimethyl‐2‐thiazolyl)‐2,5‐diphenyl‐2‐H‐tetrazolium bromide assays the estimated cell viability. (C) Dichlorodihydrofluorescein‐diacetate assays determined the reactive oxygen species levels (Scale bar = 100 μm). (D) Enzyme‐linked immunosorbent assay assays evaluated 8‐oxo‐7,8‐dihydro‐2′deoxyguanosine (8‐oxo‐dG) levels. (E) Superoxide dismutase (SOD) activity was measured by SOD detection assays. Each experiment was independently repeated three times. *p < 0.05, **p < 0.01, ***p < 0.001. DMSO, dimethyl sulfoxide; GLI2, GLI family zinc finger protein 2.

To ulteriorly demonstrate the mechanism of the interaction between ATM and miR‐200 in the DNA repair of HFF‐1 cells under HG, the cells were subjected to the transfection of ATM and miR‐200a/b/c‐3p inhibitor vectors. The cotreatment of si‐ATM reversed the promotion of miR‐200a/b/c‐3p inhibitors on ATM expression under HG conditions (Figure 4A). Also, the silencing of ATM decreased the cell viabilities of HG‐treated HFF‐1 cells with miR‐200a/b/c‐3p inhibitors transfection (Figure 4B). In addition, the suppression of miR‐200a/b/c‐3p inhibitor ROS levels was also reversed by the si‐ATM, and it induced an increase in ROS levels under HG conditions (Figure 4C). Similarly, miR‐200a/b/c‐3p inhibitor downregulated the 8‐oxo‐dG levels of HFF‐1 cells under HG, while the extra cotreatment of si‐ATM significantly increased the level of 8‐oxo‐dG in HG‐stimulated HFF‐1 cells (Figure 4D). On the contrary, the promotive effects of miR‐200a/b/c‐3p inhibitors on SOD activities of HG‐simulated HFF‐1 cells were also abolished by ATM silencing (Figure 4E). These data revealed that the silencing of ATM could abolish the positive effect of miR‐200 downregulation on cell proliferation and DNA repair under HG.

### 3.5 GLI2 overexpression promotes DNA damage repair by regulating miR‐200

**FIGURE 5:**
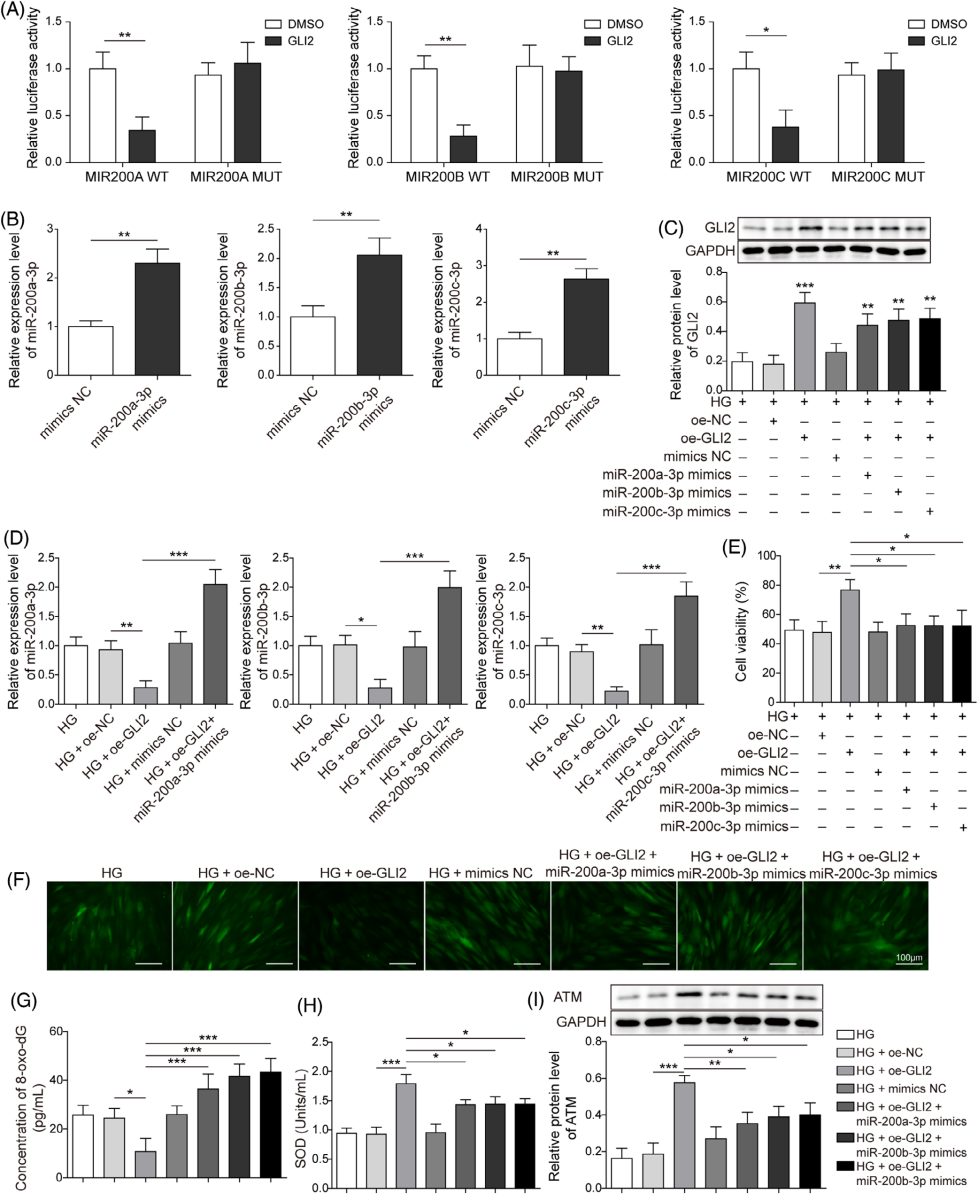
Overexpressed GLI family zinc finger protein 2 (GLI2) ameliorated the high glucose (HG)‐induced repression of DNA damage repair through the modulation of miR‐200. Human foreskin fibroblasts were treated with HG and transfected with pcDNA3.1‐GLI2, pcDNA3.1‐NC, miR‐200a/b/c‐3p mimics, and mimics negative control (NC). (A) Dual‐luciferase reporter assays determined the interaction between GLI2 and miR‐200a/b/c‐3p. (B) Quantitative real‐time polymerase chain reaction (qRT‐PCR) was used to detect the expression of miR‐200a/b/c‐3p. (C) Western blot checked GLI2 expression. (D) qRT‐PCR detected the expression of miR‐200a/b/c‐3p. (E) 3‐(4,5‐dimethyl‐2‐thiazolyl)‐2,5‐diphenyl‐2‐H‐tetrazolium bromide assays estimated cell viability. (F) Dichlorodihydrofluorescein‐diacetate assays determined ROS levels (Scale bar = 100 μm). (G) Enzyme‐linked immunosorbent assay assays evaluated 8‐oxo‐7,8‐dihydro‐2′deoxyguanosine (8‐oxo‐dG) levels. (H) Superoxide dismutase (SOD) activity was measured by SOD detection assays. (I). Western blot detected the protein expression of ataxia telangiectasia mutated (ATM). Each experiment was independently repeated three times. *p < 0.05, **p < 0.01, ***p < 0.001.

HFF‐1 cells were transfected with GLI2 and miR‐200 overexpression vectors, to validate the roles and mechanisms of GL12 and miR‐200 family in regulating cell proliferation and DNA damage repair under HG conditions. Based on the prediction of the JASPAR database (http://www.jaspar.genereg.net), there were potential GLI2‐binding sites in miR‐200a/b/c‐3p's promoter. Luciferase reporter assay analyses evidenced that GLI2 overexpression reduced the luciferase activity of the miR‐200a/b/c‐3p promoter, further validating the interaction between GL12 and miR‐200a/b/c‐3p (Figure 5A). MiR‐200a/b/c‐3p mimicked transfection and significantly promoted the relevant miR‐200a/b/c‐3p expression in HFF‐1 cells under NG, identifying the successful establishment of an overexpression cell model (Figure 5B). GLI2 expression was markedly increased in the oe‐GLI2 group, showing the successful construction of an overexpression vector (Figure 5C). Moreover, the overexpression of GLI2 repressed miR‐200 expression, while the extra overexpression of miR‐200 reversed that trend in HFF‐1 cells under HG (Figure 5D). GLI2 overexpression promoted the HFF‐1 cells vitality compared with the HG‐alone treatment group; however, miR‐200a/b/c‐3p overexpression abolished the regulation of GLI2 (Figure 5E). In addition, the overexpressed GLI2 reduced ROS and 8‐oxo‐dG levels under HG, which was also reversed miR‐200a/b/c‐3p overexpression (Figure 5F,G). GLI2 overexpression markedly enhanced SOD activities of HFF‐1 cells treated with HG, and the cotreatment of miR‐200a/b/c‐3p overexpression overturned the decrease (Figure 5H). Compared with the HG‐alone treatment group, GLI2 overexpression significantly promoted the expression of ATM under the HG circumstance, whereas the miR‐200a/b/c‐3p mimics also reversed the protein change tendency (Figure 5I). To sum up, our data indicated that the overexpressed GL12 could ameliorate the HG‐induced inhibition of cell proliferation and DNA repair via the negative regulation of miR‐200a/b/c‐3p.

### 3.6 MiR‐200 inhibition promoted DFU mice foot wound healing via enhancement of DNA damage repair

**FIGURE 6:**
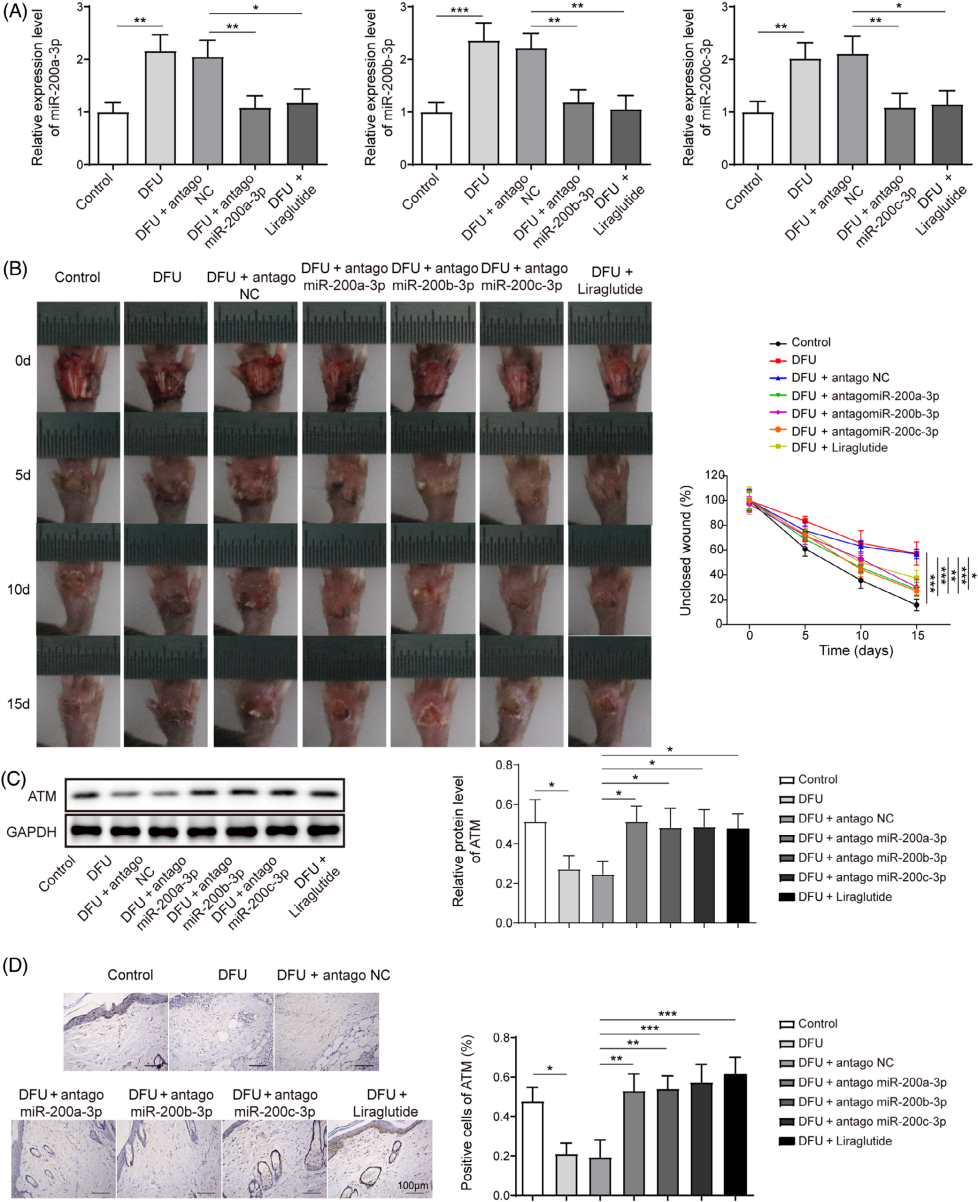
MiR‐200 inhibition promoted foot wound healing by enhancing DNA damage repair in the diabetic foot ulcer (DFU) mice model. DFU mice were administrated with antagomiR‐200a/b/c, antago‐negative control (NC), and liraglutide treatment. (A) Quantitative real‐time polymerase chain reaction detected the miR‐200a/b/c‐3p expression. (B) Wound healing in DFU mice was recorded on days 0, 5, 10, and 15. (C) Western blot examined the expression of ataxia telangiectasia mutated (ATM). (D) Immunohistochemical analysis assessed ATM expression (Scale bar = 100 μm). Each experiment was independently repeated three times. *p < 0.05, **p < 0.01, ***p < 0.001.

To validate the modulatory mechanism of miR‐200a/b/c‐3p and DNA damage repair of an in vivo model, we conducted the relevant DFU mouse model using spontaneous type 2 diabetic db/db mice, and administered miR‐200 inhibitor vector and type 2 diabetes drug liraglutide (positive control) injection in the mouse foot wound tissues. As displayed in (Figure 6A), the expression of miR‐200a/b/c‐3p was significantly upregulated in the foot wound tissues, whereas the antagomiR‐200a/b/c‐3p injection or liraglutide treatment markedly downregulated their expression. From the wound closure perspective (0–15 days' consecutive observation), the unclosed wound rates of the antagomiR‐200a/b/c‐3p or liraglutide injected DFU mice were significantly lower than the mice of the DFU group (Figure 6B). Besides, the ATM protein expression was markedly repressed in the DFU group, whereas the miR‐200a/b/c‐3p inhibition or liraglutide treatment reversed the changing trend (Figure 6C). Also, based on the immunohistochemical analysis of mice foot wound tissues, the miR‐200a/b/c‐3p inhibitor or liraglutide treatment also promoted the expression of ATM in the DFU group (Figure 6D). In conclusion, we verified that the inhibition of the miR‐200 family could significantly enhance foot wound healing via the promotion of DNA damage repair in the DFU in vivo model.

## 4 DISCUSSION

DFU is a characteristic complication of diabetes mellitus that severely damages patients' foot health and disease‐specific survival. Based on the relevant analysis, almost 10% of patients with diabetes mellitus worldwide have experienced foot ulceration.^22^ Foot skin wound healing plays a crucial role in the progression of DFU, and poor wound healing can contribute to its further deterioration. In wound healing, the proliferation, migration, and differentiation of skin fibroblasts exert vital effects on the neovascularization and wound closure of DFU recovery.^23^ Hyperglycemia always induces skin fibroblast damage, delaying wound healing. Under high‐glucose conditions, skin fibroblasts typically suffer from oxidative stress and ensuing DNA damage.^24^ Furthermore, excessive DNA damage has been ranked as one of the primary factors restraining diabetic wound healing.^25^ In this study, we found that diabetes‐induced hyperglycemia represses cell proliferation, DNA damage repair‐relevant protein expression, and wound healing in HFF‐1 cells or the DFU mouse model. Notably, we first revealed that overexpressed GL12 could repress the miR‐200 family to enhance ATM expression and ameliorate the suppression of HG‐treated HFF‐1 cell proliferation. MiR‐200 family inhibition promoted the expression of the DNA damage repair corresponding proteins and diabetic wound healing of the DFU mouse model.

MiRNAs exert vital regulatory effects on cell signaling and function in physiological and pathological processes. For instance, the abnormal expression of miRNA is extensively identified in the progression of DFU, including miR‐497 and miR‐217.^26^, ^27^ Research has shown that the miR‐200 family hurts the wound‐healing capacities of keratinocytes and embryonic stem cells, suggesting its potential regulatory role in DFU.^18^ Besides, miR‐200 family inhibition may exert a promotive effect in diabetic wound healing through the enhancement of angiogenesis.^13^ We originally revealed that the expression of miR‐200a/b/c‐3p was significantly enhanced in HG‐treated skin fibroblasts and the DFU mouse model. The high expression of miR‐200 family members was in line with the upregulated miR‐200c in the arteries of diabetic patients and mice.^28^ Moreover, for the first time, we observed that the inhibition of miR‐200a/b/c‐3p moderated HG‐induced cell proliferation and DNA damage in HFF‐1 cells and that miR‐200a/b/c‐3p inhibitors could promote wound healing in DFU mice. The observation was in line with their role in the proliferation of HG‐treated endothelial cells and wound closure of the type 2 diabetes mouse model.^29^ Collectively, our findings established the function of miR‐200 family members in enhancing the inhibition of cell proliferation, DNA damage, and wound healing delays of DFU in vitro or in vivo models.

DNA damage frequently occurs in the pathological and chemical change process, which is induced by oxidation or free radicals. Extreme DNA damage is always linked to abnormal cellular function and cell death. Especially, excessive DNA damage and inhibited DNA damage repair are also engaged with the progression of diabetes and its complications.^10^ ATM, a crucial member of signal transducers, conducts the early recruitment of DNA damage repair factors to activate repair processes.^30^ ATM deficiency always accompanies insulin resistance and diabetes or its complicating diseases.^31^ In this study, we identified that HG aggravated oxidative stress and DNA damage in the DFU skin fibroblast model and DFU mice. The protein expression of ATM was significantly repressed in the HG‐stimulated in vivo and/or in vitro model, suggesting a suppression of the DNA damage repair in DFU. Our novel findings of enhanced DNA damage and inhibited DNA damage repair were in line with the findings of previous studies on diabetic vascular smooth muscle cells.^32^ Furthermore, miRNAs can interact with their targeted proteins to regulate cellular DNA damage and relevant signaling pathways, such as the interaction between the miR‐200 family and nuclear factor erythroid 2‐related factor 2.^33^ We also found that miR‐200a/b/c‐3p possessed potential binding sites of ATM. In this study, our data first verified that miR‐200a/b/c‐3p could directly target ATM and negatively regulate ATM expression in skin fibroblasts and DFU mouse models, which originally supplemented the mechanism of action of the miR‐200 family and ATM in cell proliferation and DNA damage in DFU. The dysregulated immune modulation and impaired blood microcirculation are crucial and major factors in diabetic wound healing processes.^34^, ^35^ And miR‐200/ATM axis has been reported to modulate immune function and blood circulation in tumor and other disease models.^36^, ^37^ Our findings have provided a potentially effective pathway of the miR‐200/ATM axis to enhance diabetic wound healing, while their regulatory effects on the immune function and blood circulation in DFU need to be precisely demonstrated in the future.

GLI2 is one of the abnormally expressed transcription factors in models of diabetes and its complications. Downregulated Shh signaling and GLI2 have been found in diabetic endothelial progenitor cells.^15^ In this study, we found that HG treatment markedly repressed the GLI2 expression in the skin fibroblasts, which was similar to what was found for HG‐treated endothelial cells. Also, GLI2 overexpression can enhance cell proliferation and DNA damage repair of skin fibroblasts under HG. GLI2‐overexpressed mice possess a stronger glucose metabolism ability and insulin sensitivity than normal mice.^38^ This finding is partially consistent with our finding of GL12 overexpression‐enhanced HFF‐1 cell proliferation under HG. Congruously, the findings of a previous study indicated that Shh signaling could promote the re‐epithelialization of murine embryonic stem cells to enhance skin wound healing by negatively regulating the miR‐200 family.^18^ Similarly, we found that GLI2 overexpression negatively modulated the miR‐200a/b/c‐3p in HG‐treated skin fibroblasts. These findings supplemented and validated the interaction between GL12 and the miR‐200 family in a wound‐healing model. To the best of our knowledge, this is the first study to prove that GLI2 overexpression inhibited the harmful effects of miR‐200a/b/c‐3p on the proliferation and DNA damage repair of HG‐treated HFF‐1 cells. Collectively, we demonstrated that GLI2 alleviated the suppression of cell proliferation and DNA damage repair via the regulation of the miR‐200/ATM axis in the DFU cell model.

In conclusion, our study clarified the fact that GLI2 promoted cell proliferation and DNA damage repair by regulating the miR‐200/ATM axis to enhance wound healing in a DFU model. However, the underlying mechanism of action still requires further investigation of diabetic clinical samples. This study's findings establish the function of GLI2 and miR‐200/ATM in diabetic foot wound healing and support the hypothesis that the GLI2/miR‐200/ATM axis may be a potential target of DFU clinical treatment.

## CONFLICT OF INTEREST STATEMENT

The authors declare that they have no conflict of interest.

## Data Availability

The original data of Figure 6B Raw data from animal experiments were shown in URL: https://osf.io/6ej2z/; DOI: 10.17605/OSF.IO/6EJ2Z.
